# Advancing Interprofessional Education and Collaborative Practice: outcomes of the AFREhealth-FAIMER Student Elective Exchange Program in Health Professions Education in Africa

**DOI:** 10.3389/fmed.2025.1516156

**Published:** 2025-03-10

**Authors:** Faith Nawagi, Rashmi Vyas, Elsie Kiguli Malwadde, Shiyao Yuan, Deborah Bedoll, Prisca Olabisi Adejumo, Rory Phimister, Julie Drendall, Justin Seeling, Fatima Suleman

**Affiliations:** ^1^FAIMER, A Division of Intealth, Philadelphia, PA, United States; ^2^African Centre for Global Health and Social Transformation (ACHEST), Kampala, Uganda; ^3^Department of Nursing, University of Ibadan, Ibadan, Nigeria; ^4^School of Health Sciences, University of KwaZulu-Natal, Durban, South Africa

**Keywords:** Interprofessional Education and Collaborative Practice, Africa, virtual student elective exchange, country specific case studies, international elective

## Abstract

**Background:**

While some African health professions institutions have advanced in integrating Interprofessional Education and Collaborative Practice (IPECP) into their curricula, many still struggle with traditional, siloed training. To address this gap, the African Forum for Research and Education in Health (AFREhealth), partnering with FAIMER, a division of Intealth, developed the AFREhealth-FAIMER IPECP Student Elective Exchange Program (IPECP Program). This study assessed the IPECP competencies of participating students and gathered stakeholder perspectives on the IPECP Program.

**Methods:**

The 2018 revised Interprofessional Collaborative Competency Attainment Scale (ICCAS), containing 21 items, was used to assess student participants’ IPECP competencies before and after participating in the IPECP Program. Paired sample T-tests were run to examine if there was a significant improvement in IPECP competencies after students participated in the program, on both the total and item score levels. The study also administered online surveys to collect feedback from faculty and managers of the IPECP Program on its design, implementation, benefits, and challenges.

**Results:**

Students reported a significant improvement in IPECP competencies after participating in the program, with the mean ICCAS total score rising from 79.27 (±12.24) pre-training to 85.63 (±8.75) post-training (*t*(179) = 7.48, *p* < 0.005). All faculty participants (100%, *n* = 18) indicated that country-specific case studies facilitated teaching IPECP skills through virtual delivery. Additionally, 6 out of 18 program managers noted that this program marked their institution’s first engagement in virtual IPECP electives. All faculty (*n* = 18) and managers (*n* = 10) who responded to the survey thought the IPECP program enhanced regional collaborations and global exposure and equipped the students with cross-country IPECP skills. Internet connectivity was a cross-cutting challenge among faculty and managers given the virtual nature of the program.

**Discussion and conclusions:**

The student participants of the IPECP Program self-reported enhanced IPECP competencies, fostering an understanding of the various population health issues in multiple African countries. The findings suggest that faculty-guided, country-specific case studies may offer a viable strategy for implementing IPECP during international electives using a virtual mode of delivery.

## 1 Introduction

Interprofessional Education (IPE) in the health professions involves students from two or more health and social care professions, learning with, from, and about each other (1). Interprofessional Collaborative Practice (ICP), on the other hand, occurs when health and non-health professionals work together with patients, their families, carers, and communities to deliver high-quality care (1). Interprofessional Education and Collaborative Practice (IPECP) combines the educational aspects of IPE with the practical application of ICP to prepare professionals to work in teams to deliver patient-centered, safe, and equitable care, addressing both individual and population health needs (2).

Despite the benefits of IPECP, many healthcare professionals worldwide, including in Africa, continue to train in silos with each health profession running its activities independently without interaction with other health professions. While a few African institutions have integrated IPECP into their curricula (3, 4), the global burden of emerging, re-emerging, and new diseases highlights the need for interprofessional collaboration across country borders (5). A vivid example is the West African Ebola outbreak in 2014, which required multinational health teams to contain the epidemic (6). Nevertheless, opportunities for interprofessional learning in diverse healthcare settings remain limited in Africa.

To address this gap, the African Forum for Research and Education in Health (AFREhealth), partnering with FAIMER, a division of Intealth, developed the AFREhealth-FAIMER IPECP Student Elective Exchange Program in Health Professions Education (IPECP Program) in Africa (7). AFREhealth is an interdisciplinary group that collaborates with Ministries of Health, training institutions, and other stakeholders to enhance health care in Africa through research, education, and capacity-strengthening (8). Intealth, a Philadelphia, US-based private nonprofit, advances global healthcare education through its ECFMG and FAIMER divisions (9). FAIMER promotes excellence in International Health Professions Education through programmatic and research activities (10).

The IPECP Program aimed to prepare health professions students to work effectively in interprofessional teams and apply their knowledge, skills, and values in their future practice. This study assessed the IPECP competencies of students and gathered stakeholder insights on the IPECP Program’s design, implementation, challenges, and benefits.

## 2 Materials and methods

### 2.1 AFREhealth-FAIMER IPECP Student Elective Exchange Program (IPECP Program) - overview and description

#### 2.1.1 IPECP Program overview and student selection

A total of 13 institutions from 10 African countries participated in the IPECP Program as shown in Table 1. Twelve participating institutions were from English-speaking countries. Mozambique, although a Portuguese-speaking country, had English-proficient staff and students at its participating host institution, Lúrio University. An agreement between the host institutions, FAIMER, and AFREhealth ensured clear, reciprocal resource allocation and opportunities. AFREhealth received a grant from the National Institutes of Health (NIH), through which it contracted FAIMER to design and implement the IPECP Program. FAIMER allocated $2,800 to each host institution for internet, faculty time, and administrative costs. FAIMER’s web-based application system enabled students to view elective opportunities and submit applications, allowing institutions to manage and track applications in real-time.

**Table 1:** Participating institutions and country-specific case studies used to guide learning (*N* = 13) in AFREhealth-FAIMER Interprofessional Education and Collaborative Practice Student Elective Exchange Program in Health Professions Education in Africa (IPECP Program).

Country	Name of institution	Case study
Ethiopia	Debre Tabor University	Protein-energy malnutrition in Ethiopia.
Ghana	Kwame Nkrumah University of Science and Technology	The emergence of multi-drug-resistant bacteria “superbugs:” Implications on contemporary practice in Ghana.
Kenya	Jomo Kenyatta University of Agriculture and Technology	Care of a sick newborn in a developing country: A case study for Kenya.
Kenya	Kenyatta University Medicine	COVID-19 pandemic challenges and hopes: A case study in Kenya.
Malawi	Kamuzu University of Health Sciences	Accessibility to health-related services among children who are living with cerebral palsy in Malawi.
Mozambique	Lurio University	Community health and well-being with a view to good practices and behavior change in low-income communities in Nampula-Mozambique.
Nigeria	University of Ibadan	Cancer diagnosis in women and their quality of life in Nigeria.
Rwanda	University of Rwanda	Interprofessional approach to dog bite in Rwanda.
Uganda	Busitema University	Infection prevention and control.
Uganda	Makerere University	Targeted maternal health initiatives for reducing maternal mortality and morbidity in Uganda.
Uganda	Mbarara University of Science and Technology	COVID-19-related service delivery in Uganda.
Zambia	Lusaka School of Nursing	Adherence to COVID-19 preventive measures in Zambia’s high-density populated communities: A case of Lusaka.
Zimbabwe	University of Zimbabwe	Optimizing antiretroviral therapy adherence (O-ART) in Zimbabwe.

Each case study had a description of the case and guiding questions for the students to enable them to study the case while appreciating the various IPECP competencies described in the ICCAS. The case studies also had reference sections for the students about the case and IPECP, activities to do, and a description of the overall aim in line with IPECP.

Host institutions managed the selection of students for the IPECP Program, inviting applicants through advertisements. Selection criteria included student interest, availability for a six-week commitment, academic standing, and representation from various health professions such as medicine, pharmacy, nursing, and dentistry among others as shown in Table 2. Undergraduate clinical-year students were eligible, given their familiarity with a clinical learning environment and the ethical considerations in clinical rotations. Students applied for electives at a host institution outside their home country in groups of five, ensuring interdisciplinary representation. Faculty members teaching the students at the host institution represented at least two health professions from the students’ group. Each host institution independently accommodated one cohort from another institution, with placements scheduled according to the availability of electives and academic calendars. The interprofessional composition of each student group varied annually based on selection criteria, with a minimum requirement of students from at least two different health professions. The six-week placements involved virtual engagement in all IPECP activities.

**Table 2:** Sociodemographic characteristics of the students (*N* = 180) in the AFREhealth-FAIMER Interprofessional Education and Collaborative Practice Student Elective Exchange Program in Health Professions Education in Africa (IPECP Program).

Characteristic	Frequency (*n*)	Percentage (%)
Gender		
Female	94	52.2
Male	86	47.8
Course of study		
Anesthesia	5	2.8
Biomedical engineering	1	0.6
Biomedical sciences	1	0.6
Dentistry	7	3.9
Laboratory medicine	11	6.1
Medicine	56	31.1
Midwifery	5	2.8
Nursing	42	23.3
Nutrition	1	0.6
Occupational therapy	1	0.6
Pharmacy	30	16.7
Physiotherapy	13	7.2
Public health	2	1.1
Radiography	2	1.1
Speech and language therapy	1	0.6
Veterinary medicine	2	1.1
	**Mean**	**SD**
Age	24.54	3.85
Year of study	4.27	1.20
Duration of the undergraduate program of study	4.75	1.05

#### 2.1.2 IPECP Program curriculum

A total of 13 electives were available at the undergraduate level. The study team developed the curriculum in conjunction with IPECP experts from Yale University in the US, Makerere University in Uganda, University of Global Health Equity in Rwanda, and Stellenbosch University in South Africa. International and local expertise provided a global perspective and contextualized the curriculum to local needs. Guided by social constructivism (11) and activity learning theories (12), the curriculum for students, including the faculty training workshop, emphasized learning as a social process, fostering knowledge-building through collaboration and integration of real-world scenarios.

**Figure 1:**
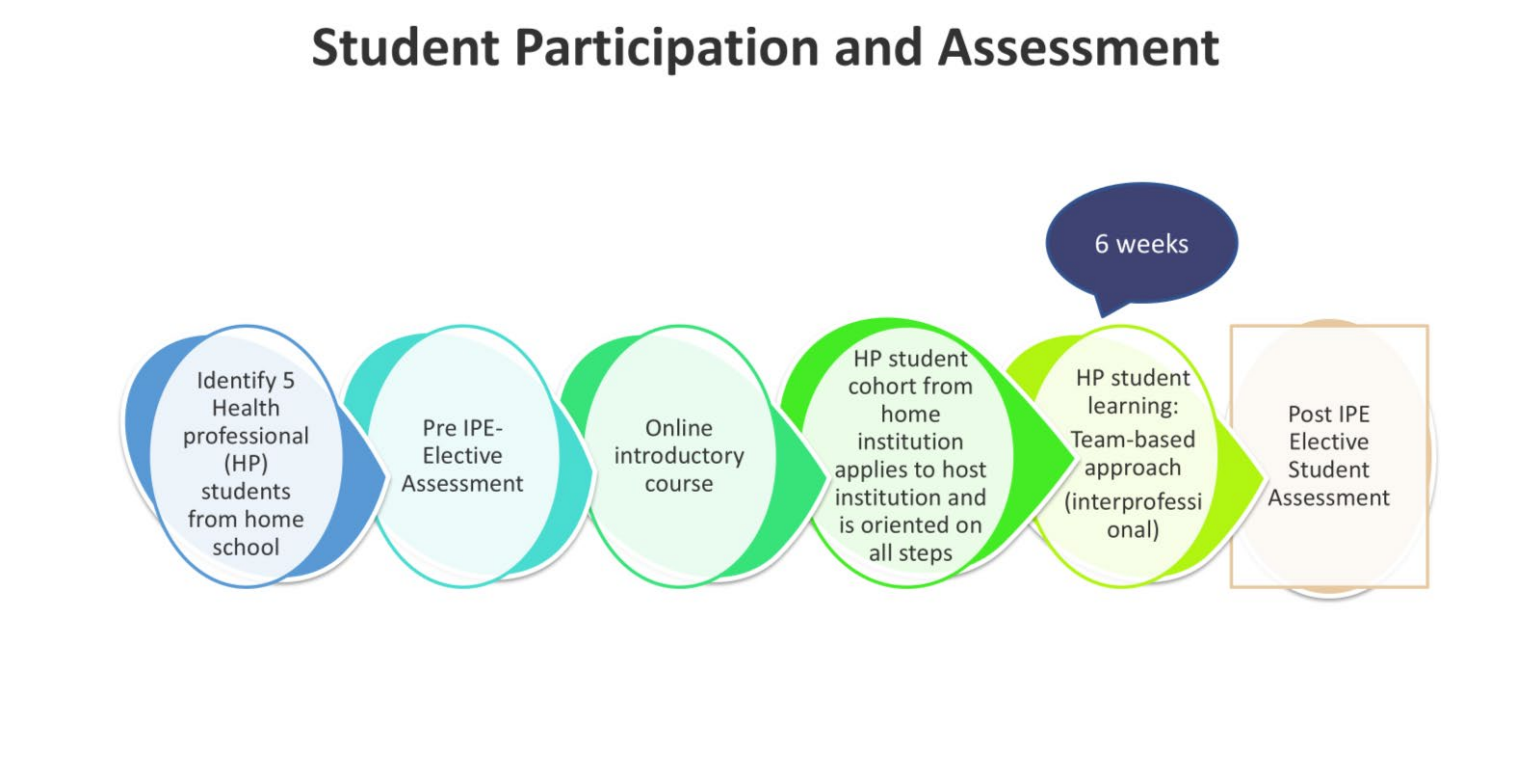
Student participation and assessment in the AFREhealth-FAIMER Interprofessional Education and Collaborative Practice Student Elective Exchange Program in Health Professions Education in Africa (IPECP Program).

The student curriculum included a virtual pre-orientation course on IPECP followed by virtual introductions to chosen host institutions. Due to COVID-19 travel limitations, virtual electives featuring country-specific case studies on population health were provided (see Table 1). The case studies were developed based on the common occurrences in the various countries in the fields of maternal and child health, public health, and epidemic disease outbreaks among others. The elective program structuring did not vary much based on the number of students at each institution or the health care system in each country. This is because each institution followed the same guidelines in terms of the number of students and how to conduct the program but only varied on what case study to use to enable learning. The activities included weekly online sessions, literature reviews, weekly progress assignments, live online interactive lectures, pre- and post-program IPECP competency self-assessments, collaborative innovation, and report writing. Figure 1 shows the flow of students’ participation and self-assessment in the IPECP Program.

Two faculty members from different professions guided the learning process at each host institution. All host institution faculty participated in a two-hour online training workshop covering IPECP competencies, program scope, teaching strategies, and assessment of IPECP in an international elective setting in Africa. FAIMER, a division of Intealth and AFREhealth, co-owned this training. It was developed and delivered with IPECP and Health Professions Education (HPE) experts from FAIMER, Makerere University, Uganda, University of Global Health Equity, Rwanda, Stellenbosch University, South Africa, and Yale University, USA. This training occurred before the IPECP Program, with annual synchronous refreshers for ongoing skill reinforcement. The virtual component was regional within Africa, and each cohort rotated through a host institution outside their home country.

### 2.2 Study design and measures

This analytic cross-sectional study examined students’ self-assessed IPECP competencies before and after their participation in the IPECP Program from 2021 to 2023. The 2018 revised Interprofessional Collaborative Competency Attainment Scale (ICCAS) (13) was used to self-assess students’ IPECP competencies. The ICCAS, is a validated tool and includes 21 statements intended to measure the self-reported competencies of interprofessional care in interprofessional education programs (14, 15). Twenty statements measured IPECP domains, such as teamwork, interprofessional communication, shared values, and ethics of interprofessional collaboration on a 5-point Likert scale (1 = “Poor” to 5 = “Excellent”), with the total scores ranging from 20 to 100. The 21st statement measured the students’ general ability to collaborate interprofessionally as compared to their ability before the IPECP Program, on a reverse 5-point Likert scale where 1 = “Much better now” and 5 = “Much worse now.”

The study team developed two surveys: one for faculty (Faculty Survey) and another for managers (Manager Survey). These surveys were designed due to the lack of existing tools and literature for assessing the implementation of Interprofessional Education and Collaborative Practice (IPECP) during international electives among faculty and managers on a global scale. Drawing on prior experience in conducting evaluation studies in international elective programs across various institutions and countries in Africa (16, 17), the team ensured the surveys were contextually relevant. The surveys were piloted among FAIMER Africa staff, who worked closely with both faculty and managers and had a deep understanding of their respective roles. This phase aimed to establish the surveys’ rigor and usability, ensuring clarity and alignment with the intended objectives. Revisions were made post-pilot to enable alignment with the intended research objectives. The questions to the managers were mainly operational and one addressed IPECP skills development. This was added because the managers were provided with an orientation on the IPECP definition, competencies, and how to aid the operational implementation of the various IPECP electives, as part of their training. Thus, their views on IPECP are key yet often missed in Health Professions Education program evaluation.

The Faculty Survey gathered the insights of the faculty, using a 5-point Likert scale under the following sections: (1) overall program functionality and virtual implementation, (2) teaching resources and tools, (3) communication methods, (4) student learning experience, (5) faculty skills and commitment to IPECP continuation, and (6) program benefits. An open-ended question in the Faculty Survey was used to elicit the challenges of the IPECP Program design and delivery.

The Manager Survey gathered the insights of the managers under the following sections using either a 5-point Likert scale or a Yes/No scale: (1) students’ skill development, (2) reciprocity and partnerships, (3) virtual participation and preferred delivery, (4) program benefits, (5) getting academic credit for participation in the IPECP Program, and (6) challenges of the IPECP Program design and delivery.

### 2.3 Ethics considerations

Ethics clearance was granted by the Mulago Hospital Research and Ethics Committee (MHREC-2024-156) and the Uganda National Council for Science and Technology (HS4461ES). Administrative approval was secured through Memoranda of Understanding (MOU) between FAIMER, AFREhealth, and the 13 participating institutions. Informed consent was sought from every participant and password protection of the database using the Intealth data privacy policy was done to observe confidentiality.

### 2.4 Study participants

The participants of this study included all the faculty, students, and managers of the IPECP Program. The managers were the administrative staff with administrative education background at each university. The managers usually handle the operational, instructional design, technical, and administrative implementation of international elective programs at each participating university institution. Study participants were all invited to participate online via their email addresses. Online consent was sought from the participants by reading the consent statement, and if they agreed to participate, they would click, “Yes.” This would enable them to proceed to the survey section. The email to all participants was sent by the FAIMER Africa administrator with the link to the survey.

### 2.5 Data collection and analysis

The ICCAS scale was administered online via Microsoft Forms to each student cohort from 2021 to 2023 at the beginning and the end of the IPECP Program as a mandatory part of the curriculum. Student participation in the program and assessment is shown in Figure 1. In each administration, the ICCAS scale was open for 1 month, and two reminders were sent. Faculty and Manager Surveys were administered online via Microsoft Forms to the faculty and managers in May 2024 voluntarily. The surveys were available for 2 months, with weekly email reminders sent.

For univariate analysis of participant characteristics, frequencies, proportions, and measures of central tendency, i.e., the mean, were used. Paired-sample t-tests were conducted to assess the statistical significance of students’ ICCAS scores pre- and post-IPECP Program participation. Data analysis was done using SPSS Statistics version 29 (IBM Corp, Armonk, New York).

Descriptive statistics (for closed-ended questions) and content analysis (18) (for open-ended questions) were utilized in the Faculty Survey to identify challenges they faced in the IPECP Program. Content analysis was manually done deductively. Themes were predetermined, followed by reviewing the texts, identifying codes, and quantifying the findings from the themes (18).

## 3 Results

### 3.1 Sociodemographic characteristics of the student participants of the IPECP Program

All 180 students from the IPECP Program (2021–2023) completed the ICCAS scale, achieving a 100% response rate. The majority of the students (80.5%, *n* = 145) reported no prior experience with IPE at their training institutions while a few, (19.5%, *n* = 35) reported prior experience with IPE. The participants represented 16 health professions, with medicine (31.1%, *n* = 56) and nursing (23.3%, *n* = 42) being the most common, as shown in Table 2.

### 3.2 Students’ IPECP competency

There was an increment in the total mean score of the students, 85.63 (±8.75) on the post-ICCAS score compared to the mean pre-ICCAS score of 79.27 (±12.24). The paired sample *t*-test showed a statistically significant improvement in the IPECP competency of the students compared to their pre-IPECP Program baseline (*t*(179) = 7.48, *p* < 0.001). Follow-up analyses on pre-and post-IPECP Program scores on each of the 20 items in the ICCAS scale were performed using Bonferroni correction adjusted alpha levels of 0.0025 (0.05/20 items). A *p*-value less than 0.0025 was considered statistically significant. The results (see Table 3) indicate a statistically significant increase in post-program ICCAS scores in most of the 20 items on the ICCAS scale. No significant difference was detected for the items *Include the patient/family in decision-making* and *Be accountable for my contributions to the IP team* after the Bonferroni correction.

**Table 3:** Students’ ICCAS total and item mean scores pre- and post-participation in the AFREhealth-FAIMER Interprofessional Education and Collaborative Practice Student Elective Exchange Program in Health Professions Education in Africa (IPECP Program) (*N* = 180).

Interprofessional Collaborative Competency Attainment Scale (ICCAS)	Pre-IPECP program score Mean (±SD)	Post-IPECP program score Mean (±SD)	*t*(df)	*p* value
Total score	79.27 (±12.24)	85.63 (±8.75)	7.48 (179)	< 0.001*
Promote effective communication among members of an interprofessional (IP) team	4.03 (±0.83)	4.47 (±0.61)	5.68 (179)	< 0.001*
Express my ideas and concerns without being judgmental	4.07 (±0.93)	4.53 (±0.67)	5.42 (179)	< 0.001*
Provide constructive feedback to IP team members	4.10 (±0.89)	4.47 (±0.64)	4.52 (179)	< 0.001*
Express my ideas and concerns in a clear, concise manner	4.14 (±0.89)	4.46 (±0.96)	3.64 (179)	< 0.001*
Seek out IP team members to address issues	4.03 (±0.94)	4.45 (±0.69)	5.04 (179)	< 0.001*
Work effectively with IP team members to enhance care	4.34 (±0.77)	4.56 (±0.63)	3.17 (179)	< 0.001*
Learn with, from, and about IP team members to enhance care	4.38 (±0.72)	4.62 (±0.55)	3.61 (179)	< 0.001*
Identify and describe my abilities and contributions to the IP team	4.05 (±0.83)	4.43 (±0.69)	4.88 (179)	< 0.001*
Be accountable for my contributions to the IP team	4.32 (±0.82)	4.50 (±0.64)	2.39 (179)	0.009
Understand the abilities and contributions of IP team members	4.13 (±0.80)	4.51 (±0.65)	5.17 (179)	< 0.001*
Recognize how others’ skills and knowledge complement and overlap with my own	4.23 (±0.77)	4.56 (±0.68)	4.7 (179)	< 0.001*
Use an IP team approach with the patient to assess the health situation	4.06 (±0.98)	4.49 (±0.71)	5.27 (179)	< 0.001*
Use an IP team approach with the patient to provide whole-person care	4.07 (±0.93)	4.46 (±0.73)	4.78 (179)	< 0.001*
Include the patient/family in decision-making	4.20 (±0.99)	4.41 (±0.79)	2.21 (179)	0.014
Actively listen to the perspectives of IP team members	4.43 (±0.75)	4.68 (±0.51)	4.11 (179)	< 0.001*
Take into account the ideas of IP team members	4.34 (± 0.79)	4.65 (±0.55)	4.40 (179)	< 0.001*
Address team conflict in a respectful manner	4.19 (±0.88)	4.47 (±0.71)	3.28 (179)	< 0.001*
Develop an effective care plan with IP team members	4.12 (±0.863)	4.55 (±0.67)	5.37 (179)	< 0.001*
Negotiate responsibilities within overlapping scopes of practice	4.05 (±0.87)	4.36 (±0.06)	3.96 (179)	< 0.001*

Each item is scored on a scale of 1–5. The total score summed up the scores of all 20 items. The probability level to establish a statistical relationship is set at < 0.05 for the total mean score comparison, and < 0.0025 for the item mean score comparison. *t* is the *T* value and the df is the degree of freedom related to the sample size.

* Denotes statistical significance.

The last question, i.e., the 21st question in the ICCAS tool assessed students’ ability to collaborate interprofessionally as compared to their ability before the IPECP Program. After participating in the IPECP Program, 81.6% (*n* = 147) of students perceived that their ability to collaborate interprofessionally was better, 2.2% (*n* = 4) saw no change, and 16% (*n* = 29) perceived that their ability to collaborate interprofessionally was worse than before participating in the IPECP Program.

### 3.3 Feedback from faculty of the IPECP Program

#### 3.3.1 Faculty characteristics

**Figure 2:**
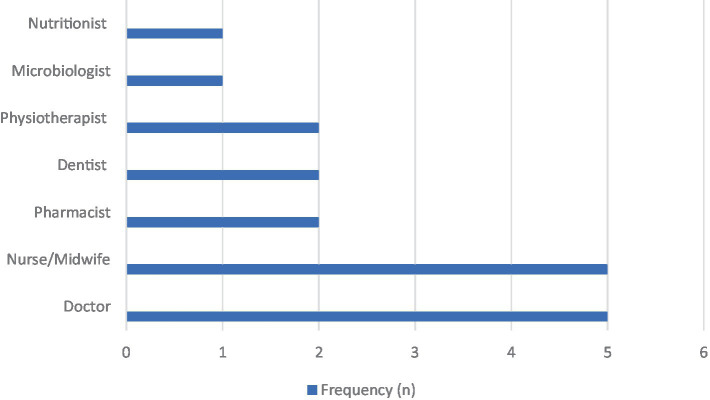
Distribution of the faculty participants who responded to the survey, by professional discipline (*N* = 18), regarding the AFREhealth-FAIMER Interprofessional Education and Collaborative Practice Student Elective Exchange Program in Health Professions Education in Africa (IPECP Program).

Out of the 27 faculty members who participated in this program, 18 responded to the survey, yielding a 67% response rate. Figure 2 depicts the professional distribution of the participating faculty. More than half of the faculty respondents were nurses/midwives (*n* = 5 out of 18) and physicians (*n* = 5 out of 18). More than half of the faculty (*n* = 10 out of 18) had prior experience and training in IPE while (*n* = 8 out of 18) did not have prior training. Nonetheless, the online workshop enabled all faculty to be equipped with the skills required to guide students’ learning.

#### 3.3.2 Faculty perspectives of the IPECP Program

Faculty perspectives of the IPECP Program Survey are presented in the following sections. The results are a representation of the number of faculty respondents and not all the faculty that participated in the program.

##### 3.3.2.1 Overall program functionality and virtual implementation

All (*n* = 18) of the faculty respondents agreed or strongly agreed that the virtual approach was functional for the program’s implementation during and after the COVID-19 pandemic. The majority (*n* = 17 out of 18) of the respondents agreed or strongly agreed that the pre-program faculty training workshops focusing on IPECP teaching, assessment skills, and program goals provided them with suitable preparation for the program, and one faculty member responded as neutral. The majority (*n* = 14 out of 18) of respondents agreed or strongly agreed that the 6-week duration was adequate for the smooth operationalization of the IPECP Program, while three faculty members were neutral, and one disagreed.

##### 3.3.2.2 Teaching resources and tools

All (*n* = 18) respondents agreed or strongly agreed that the country-specific case studies were useful learning tools for teaching IPECP skills. All (*n* = 18) respondents agreed or strongly agreed that the creation of teaching plans at each institution enabled well-organized and synchronized learning activities. The majority (*n* = 17 out of 18) of the respondents agreed or strongly agreed that the Zoom platform was adequate for synchronous learning, demonstrating the program’s adaptability to a virtual learning format, and one faculty member responded as neutral.

##### 3.3.2.3 Communication methods

The majority (*n* = 17 out of 18) of the respondents agreed or strongly agreed that communication via WhatsApp was considered adequate for communication between faculty and students, and only one faculty member responded as neutral. All (*n* = 18) found email communication adequate in sharing learning materials and introducing students to host institution faculty.

##### 3.3.2.4 Student learning experience

Most respondents (*n* = 16 out of 18) agreed or strongly agreed that the pre-orientation and 6-week virtual elective format was adequate and that the program structure effectively met the learning objectives, and two faculty members responded neutrally. The majority of the respondents (*n* = 17 out of 18) agreed or strongly agreed that the assessment approaches including using the ICCAS scale pre- and post-IPECP Program, report submissions, and virtual group presentations, were effective; one faculty member responded neutrally to this.

##### 3.3.2.5 Faculty skills and commitment to IPECP continuation

All (*n* = 18) of the faculty respondents agreed or strongly agreed that they could mentor and teach IPECP to more students after teaching in the IPECP Program. They would also like to see their institutions continue to participate in the IPECP Program.

##### 3.3.2.6 Program benefits

All (*n* = 18) respondents agreed or strongly agreed that the IPECP Program contributed to (1) promoting regional collaboration on various health issues; (2) equipping health professions students in Africa with cross-country regional IPECP skills; (3) breaking student elective barriers that exist in various countries in Africa, especially during the COVID-19 pandemic; and (4) strengthening of intra-Africa institutional partnerships. Fourteen respondents agreed or strongly agreed that the IPECP Program contributed to enhancing international exposure to African students through exchanges; four faculty members responded neutrally to this.

##### 3.3.2.7 Challenges

Content analysis of open-ended responses revealed that many of the faculty respondents (*n* = 12 out of 18) reported challenges related to the virtual mode of delivery, primarily due to unstable internet connectivity. To manage this, participants often turned off videos to optimize connectivity, and extended session times to complete courses.

### 3.4 Feedback from program managers of the IPECP Program

Out of the 13 program managers, 10 responded to the Manager Survey, yielding a 77% response rate. The Manager Survey data reflected the experiences of the managers who responded to the survey of the IPECP Program and not all the managers under the following sections.

#### 3.4.1 Virtual participation and preferred delivery

Nine managers agreed or strongly agreed that the online application management system was viewed as adequate in enabling the centralization of applications for outgoing and incoming students while enabling easy visibility of elective opportunities at the various training institutions. More than half (*n* = 6 out of 10) of the program managers reported that it was their institution’s first time participating in virtual IPECP electives. Despite positive experiences with a virtual mode of delivery, the majority (*n* = 9 out of 10) reported that they would have preferred a blended approach that incorporated both online and in-person interaction after the COVID-19 pandemic.

#### 3.4.2 Students’ skills development

All the IPECP Program managers who responded to the survey (*n* = 10 out of 10), agreed or strongly agreed that the IPECP Program enabled students to gain IPECP skills.

#### 3.4.3 Reciprocity and partnerships

Most program managers (*n* = 9 out of 10) reported that the multilateral agreement enabled reciprocity, with equal opportunities being available to all students and participating institutions. Most (*n* = 9 out of 10) also reported that more regional institutional partnerships were formed through participation in the program.

#### 3.4.4 Program benefits

All the IPECP Program managers (*n* = 10) agreed or strongly agreed that the IPECP Program contributed to global exposure for the students, and enabled students to acquire knowledge that is applicable back home given the similarity in disease and health systems among various African countries. The program benefits reported by the managers were similar to those reported by the faculty.

#### 3.4.5 Degree academic credit

Although the IPECP Program offered a very imperative learning experience for the students, almost all the program managers (*n* = 9 out of 10) reported that the students did not gain academic credit toward their final university degree programs for their participation.

#### 3.4.6 Challenges

The challenges reported by the managers included internet connectivity (*n* = 9 out of 10), students not being responsive on time (*n* = 5 out of 10), and faculty not being responsive on time (*n* = 1 out of 10).

## 4 Discussion

This study examined students’ self-assessed IPECP competencies before and after their participation in the IPECP Program and gathered feedback from program faculty and managers on the IPECP Program’s design, implementation, benefits, and challenges.

Results from the students’ self-assessed post-program ICCAS scores compared to pre-program ICCAS scores were statistically significant, indicating that the virtual program design and country-specific case studies enhanced students’ IPECP competencies. Specifically, students reported improved competencies in roles and responsibilities, values and ethics, communication, teamwork, patient-centered care, and conflict resolution. The IPECP Program fostered interdisciplinary collaboration, enhanced students’ knowledge for application in African institutions, and promoted transcultural understanding and future partnerships. These findings align with previous studies (17–19) which reported that regional electives in Africa enhance knowledge relevant to students’ home countries.

The findings further evidence that international elective spaces could be a viable ground to advance innovation in Health Professions Education, including IPECP. This is similar to the findings in another study by Estevez (20), in which international electives were found to be a viable platform to advance interprofessional education but were often not utilized. Furthermore, the findings of this study confirm the positive perception of interprofessional education among faculty (20, 21) and students (22) in the African region.

Most managers at participating institutions viewed this program as the first of its kind, as international electives are typically health professional-specific, connecting students with their professional counterparts and faculty at host institutions (23). The positive survey results from the IPECP Program can create confidence that international electives can effectively enhance IPECP skills. However, IPECP expertise remains limited among faculty in many African health training institutions (24). This was addressed in the IPECP Program by providing training to faculty prior to the start of the program, which was identified by the faculty as a key support for teaching. A framework for IPECP implementation in sub-Saharan Africa has been developed that incorporates faculty training as an important element and has the potential for adoption throughout Africa and globally (25).

The program’s virtual mode of delivery enabled more students to participate affordably in international electives. The cost of travel, accommodation, and meals often impedes some African students from participating in international electives (19), despite the added value of learning they offer. The IPECP Program enabled institutions to leverage the virtual mode of delivery to foster international elective learning within Africa and thus address one of the biggest barriers to participation in international electives by African health professions students.

While the IPECP Program received positive feedback from students, faculty, and managers, challenges emerged, particularly related to language barriers at a non-English-speaking host institution. English-speaking students struggled with communication, affecting collaboration. Similar challenges were reported in other international placements in sub-Saharan Africa (23). English-fluent faculty later provided additional support and extra session time, which should be integrated from the program’s outset for early intervention. The impact of this on the students’ acquisition of IPECP competencies was not studied; however, it is possible that it could affect their understanding, learning, and acquisition of the intended IPECP competencies.

Faculty and program managers cited poor internet connectivity as a challenge. Although managed by turning off videos and extending sessions, it still reduced engagement and hindered instructors’ ability to monitor understanding. Internet connectivity issues are common in online training across Africa (26); nevertheless, initiatives like Eduroam have improved the bandwidth at various training institutions (26, 27). Besides internet connectivity issues, the limited focus on IPECP in African health professions curricula remains a challenge, requiring attention at accreditation and quality assurance levels (28).

Despite the economic advantage of the virtual mode of delivery, there was still a preference for a blended mode of delivery to enable real-time physical experiences. While the blended mode of delivery is key in advancing learning, especially in the clinical domain (29), institutions should be able to choose the virtual, blended, or physical mode of delivery based on the resources available to enable more students to participate in international elective placements.

Program managers reported that most of the students did not gain credit toward their university degree for their participation in the IPECP Program. This is not uncommon for international electives in various health professions training institutions, where most of the time, students do not gain credit for participation (19). Some African institutions have added international electives to the curriculum, recognizing the valuable learning experiences and knowledge gained (29, 30, 31). However, more effort needs to be made to achieve this goal at the various training institutions in Africa.

The study has some limitations. First, it was conducted among AFREhealth member institutions in sub-Saharan Africa, making findings relevant to similar settings but not generalizable to all of Africa. Second, the sample size of the faculty and managers was small and may be seen as a limitation in reporting the findings quantitatively. Nevertheless, the triangulation of evidence by surveying everyone was key despite the faculty and managers being a small sample size thus enabling all-around feedback among all stakeholders. In addition, it is possible to study a small sample size especially when the primary focus is mainly to establish the relative proportions of the categories within the data set as in this study, and not comparisons within categories and other data sets (32). However, a mixed methods approach would be more appropriate with a bigger sample size. Third, the ICCAS scale’s reverse scaling on the last 21st question that measured students’ overall ability in interprofessional collaboration could have caused response bias, and self-reporting may have led to acquiescence bias. Looking at other studies using the ICCAS scale to assess IPE competencies among health professional students, they only utilized the first 20 items of the scale and often exclude the findings on the last 21st question (33–36) despite it having been added to the revised ICCAS 2018 version to enable learners to evaluate their overall experience in the IPE training intervention they have participated in (37). This could be due to the fact that the developers of the scale only provided the scale to use, with hardly any mention of the limitations it could have (37). Furthermore, there is hardly any literature documenting the challenge of response bias given the reverse scaling of the 21st question compared to the rest of the 20 Items.

Reflection time and a validated tool to assess competencies were used to mitigate these biases, with additional input from faculty and managers for cross-validation. The study emphasized IPECP competency gains and stakeholder perspectives. For the students, given the response bias that may come from self-assessment with the ICCAS tool, more assessment methods were adopted, including group presentations by each cohort of students to the teaching faculty at the end of the program and submission of reports. These were specific to each group since the case studies used varied and thus the findings were not reported as an aggregate. However, future research should include more objective assessments of competencies and a longitudinal study to evaluate the IPECP Program’s long-term impact. Furthermore, a study to describe how the various demographic characteristics including English proficiency vary with the IPECP competency gains and stakeholder perspectives with a bivariate and multivariate analysis approach would provide valuable insights with a bigger sample size.

## 5 Conclusion

This evaluation of the AFREhealth-FAIMER IPECP Student Elective Exchange Program in Africa indicates it has enhanced students’ interprofessional education and collaborative skills while exploring diverse population health issues across African countries. Faculty-guided, country-specific case studies can support IPECP in virtual international electives, offering a cost-effective solution for African health professions institutions and students. While this study used a self-assessment tool, more objective assessments are required to enable quality assurance and improvement of IPECP assessment methods during international electives. Long-term longitudinal studies would enable researchers to track the various behavioral changes of the participants throughout their careers, given that IPECP has a lot of behavioral aspects that often require ample time to appreciate the change.

## Data Availability

The original contributions presented in the study are included in the article/supplementary material, further inquiries can be directed to the corresponding author.
